# Delivery of selenium using chitosan nanoparticles: Synthesis, characterization, and antioxidant and growth effects in Nile tilapia (*Orechromis niloticus*)

**DOI:** 10.1371/journal.pone.0251786

**Published:** 2021-05-18

**Authors:** Juliana M. Araujo, Rodrigo Fortes-Silva, Cícero C. Pola, Fernando Y. Yamamoto, Delbert M. Gatlin, Carmen L. Gomes

**Affiliations:** 1 Department of Animal Science and Veterinary Medicine, Federal University of Bahia, Salvador, Bahia, Brazil; 2 Laboratory of Feeding Behavior and Fish Nutrition, Center of Agricultural, Environmental and Biological Sciences, Federal University of Bahia, Cruz das Almas, Bahia, Brazil; 3 Department of Mechanical Engineering, Iowa State University, Ames, Iowa, United States of America; 4 Department of Wildlife and Fisheries Sciences, Texas A&M University, Texas, United States of America; Kafrelsheikh University, EGYPT

## Abstract

This study aimed to elucidate the effects of selenium-loaded chitosan nanoparticles used as a dietary supplement on Nile tilapia (*Oreochromis niloticus*) antioxidant and growth responses. First, chitosan-based nanoparticles containing selenium (Se) were synthesized using the ionotropic gelation method and their physicochemical characteristics, controlled release profile, and antioxidant activity properties were investigated. Thereafter, the effects on glutathione peroxidase and antioxidant activities (by radical scavenging activity), growth, and whole-body composition of Nile tilapia were evaluated when they were fed with Se-loaded chitosan nanoparticles and compared with other selenium dietary supplements. Se-loaded chitosan nanoparticles showed high entrapment efficiency (87%), spherical shape, smooth surface, and broad size distribution. The controlled release of Se consisted of an initial burst followed by a gradual release over 48 h. Se-loaded nanoparticles presented significantly higher antioxidant activity compared to free Se. A 60-day feeding trial was conducted to compare the effects of supplementing different dietary Se sources, including selenomethionine (as organic source), sodium selenite (as inorganic source), and Se-loaded chitosan nanoparticles (Se-Nano and Se-Nano x1.5) on antioxidant and growth responses of Nile tilapia. A basal diet without Se supplementation was used as the control. The dietary supplementations with different Se sources (free and encapsulated selenium) lead to significant improvements in final weight and feed efficiency of Nile tilapia fingerlings. However, dietary treatments did not affect whole-body protein and lipid content. Diets containing Se-Nano and Se-Nano x1.5 were more effective than sodium selenite and selenomethionine in preventing oxidative stress and improving antioxidant activity in Nile tilapia. Overall, Se-loaded nanoparticles presented a great potential as an efficient source for delivering dietary Se to Nile tilapia, directly affecting the growth performance, feed efficiency, oxidative stress, and antioxidant activity of this species.

## Introduction

Selenium (Se) appears to play a dichotomous role in living organisms because it can act as both a nutrient and a toxicant [1] with a narrow margin of tolerance. The importance of Se in fish and seafood nutrition has received significant attention with numerous studies to evaluate its role and optimum dietary level [1–5]. Se assists with protein repair at the DNA level, being an important part of the oxidative-stress defense mechanism [2], which have been reported to improve stress resistance, increase survival, and reduce branchial anomalies of gilthead seabream (*Sparus aurata*) [3, 4]. This element helps with mineralization and bone formation processes, and prevents the oxidative damage of cytoplasmatic structures, because it is an integral part of the enzyme glutathione peroxidase [3]. Se diet supplementation also can help with the reduction of reactive oxygen species in the liver and kidney of Nile tilapia (*Oreochromis niloticus*) [1]. As a nutrient, the dietary Se requirement of fish ranges between 0.1–0.5 mg g^-1^ dry weight (d.w.), and its beneficial effects are well established [1]. Currently, inorganic Se (i.e., sodium selenite) is the most common source of Se used to supplement fish diets [5]. However, organic Se, as an amino acid chelate (i.e., selenomethionine), has shown higher bioavailability than sodium selenite [6]. In addition, the organic Se form has been shown to influence body lipid reserves and growth rate of gilthead seabream [3]. In addition to its well-known benefits in fish diets, the application of organic Se is limited due to its susceptibility to environmental conditions, such as pH and ionic strength, which decreases its water solubility, promoting aggregation and reducing its bioavailability [7, 8]. Limitations associated with environmental conditions can be overcome with the use of encapsulation strategies (*viz*. microencapsulation).

Microencapsulation has been used as a vehicle of nutrient delivery, reducing the leaching of nutrients and improving their solubility in water [9]. Furthermore, microencapsulation provides controlled release of nutrients, potentially reducing toxic effects, increasing bioavailability, and, consequently, improving aquaculture feed efficacy and quality [9, 10]. The use of microencapsulation as an alternative to improve nutrient availability has been demonstrated in the past in numerous studies [10–13]. Aragão et al. [11] showed that encapsulation using fish gelatin and Arabic gum as encapsulant materials prevent excessive taurine leaching from *Senegalese sole* feeds. In another study, white shrimp (*Litopenaeus vannamei*) fed with a blend of organic acids microencapsulated with stearin showed increased fish growth, dry matter production, and phosphorus digestibility [12]. Recently, the use of casein and dextrin to encapsulate oil was shown to improve the immune function and intestinal health of Nile tilapia fed a high-fat diet [13]. Additionally, nutrient stability in water is one of the critical factors that determines the suitability of nutrient supplements for fish larvae, as observed by Ozkizilcik et al. [10] when studying a complex microencapsulated diet for striped bass (*Morone saxatilis*). Different types of synthetic and natural polymers have been applied to produce particles for controlled release and prolonged stability of their contents [14].

Chitosan is a naturally derived, biocompatible, and biodegradable polymer that is commonly applied for microencapsulation of active components. This polymer is obtained by the deacetylation of chitin, a polysaccharide found in crustacean shells, insects, and fungi [15]. The protonation of chitosan amino groups provides chitosan with interesting properties such as antimicrobial activity, pH-responsiveness, and bioadhesiveness, which have been extensively explored for medical and pharmaceutical applications, including targeted drug delivery and edible coatings [15, 16]. Recently, when used as a diet supplement (0.25 chitosan kg^-1^), chitosan boosted resistant of rainbow trout (*Oncorhynchus mykiss*) against environmental stress and enhanced immunological parameters [17]. In aquaculture, chitosan has been used as a carrier for different types of DNA and vaccine delivery systems for fish [9, 18–22]. Moreover, the use of chitosan as encapsulant material for Se has been described in a few studies [23–25]. However, there are no studies concerning the effects of encapsulated Se on growth and antioxidant responses of fish such as Nile tilapia (*Oreochromis niloticus*). Therefore, Se microencapsulation using chitosan seems to be a viable alternative and presents great application potential in aquaculture.

Hence, this study aimed to produce chitosan-based nanoparticles to encapsulate Se and characterize their physicochemical and controlled release properties. The effects of different forms of selenium delivery on fish antioxidant and growth responses were also investigated in a comparative feeding trial. Specifically, the effects on glutathione peroxidase and antioxidant activities (by radical scavenging activity), growth and body composition of Nile tilapia (*Oreochromis niloticus*) fed with chitosan-based nanoparticles containing selenium were compared with other dietary selenium sources. Nile tilapia (*Oreochromis niloticus*) was selected for this study since it has become the second most cultured fish in the world, farmed in over 100 countries [26]. Moreover, this promising Se-loaded chitosan nanoparticles can be used in production of other fish, which reinforces its important contribution to aquaculture, considering that fish production is predicted to continue increasing to help meet the global demand for seafood [27].

## Materials and methods

### Materials

Low molecular weight chitosan (85% deacetylation, Mw 50–190 kDa), sodium selenite (Na_2_SeO_3_), trehalose, 2,3-diaminonaphthalene (DAN), phosphate buffer saline (PBS), 2,2-diphenyl-1-picrylhydrazyl (DPPH), sodium hydroxide, and hydrochloric acid were purchased from MilliporeSigma Co. (St. Louis, MO, USA). Sodium triphosphate (TPP), ethylenediaminetetraacetic acid disodium salt (EDTA, 5 M solution) and Coomassie plus (Bradford) assay kit were obtained from Thermo Fisher Scientific (Waltham, MA, USA). Glutathione peroxidase assay kit was obtained from Trevigen (Gaithersburg, MD, USA). Acetic acid, cyclohexane, 2,2-azinobis (3-ethylbenzothiazoline-6- sulfonic acid) diammonium salt (ABTS), nitric acid, and hydrogen peroxide were purchased from VWR International (Radnor, PA, USA).

### Chitosan-selenite nanoparticle preparation

Chitosan selenite nanoparticles were prepared by the ionotropic gelation method (S1 Fig, see S1 File) following previous protocols [23–25, 28]. Initially, chitosan solution (2.5 mg mL^-1^, pH 4.6) was prepared by dissolving chitosan in 1% (w/v) acetic acid solution and stirring for 6 hours at room temperature. Next, sodium selenite aqueous solution (0.6 mg mL^-1^) was added dropwise to the chitosan solution while stirring at 500 rpm for 2 hours. Then, the tripolyphosphate (TPP) aqueous solution (0.25 mg mL^-1^) was added dropwise into the chitosan/sodium selenite suspension and stirred for 2 hours to form the particles. Next, the particle suspension was sonicated (Sonic Wave CD-2800 ultrasonic cleaner, 60 W, Newtown, CT, USA) for 15 min, followed by homogenization (Ultra-Turrax T25 Basic IKA-Words Inc., Wilmington, NC, USA) at 13,000 rpm for 30 min and then allowed 24 hours to equilibrate at room temperature. After synthesis, Se-loaded chitosan nanoparticles were concentrated in rotaevaporator (Rotavapor R-300, Buchi, New Castles, DE, USA) and purified through ultrafiltration using a Millipore Labscale™ TFF system fitted with a 50 kDa molecular weight cutoff (Pellicon XL-Millipore, Millipore Co., Kankakee, IL, USA). Prior the freeze-drying process, (D+)-trehalose was added to the nanoparticles suspension as a cryoprotectant in a ratio of 1:1 (w/w chitosan). Finally, nanoparticles were frozen at -80°C overnight and freeze-dried at -50°C and 12 Pa for 48 h in a Labconco FreeZone 4.5 unit (Labconco, Kansas City, MO, USA). Unloaded nanoparticles, used as control, were prepared following the same procedure, except for the addition of sodium selenite. Dried nanoparticles were collected and stored in a desiccator at -20°C until further analyses.

### Nanoparticles characterization

#### Entrapment efficiency (EE %)

The entrapment efficiency was measured using the method described by Luo et al. [23] with modifications. Briefly, 5 mg of Se-loaded nanoparticles were suspended in 5 mL of water and mixed thoroughly. The suspensions were agitated at 80 rpm for 48 h protected from light at room temperature. Next, the suspensions were vortexed for 10 s and filtered using 0.2-μm cellulose syringe filter (MilliporeSigma) to remove undissolved chitosan and TPP. Then, 2 mL of 0.05 M EDTA and 2 mL of 0.1% (w/v) DAN (2,3-diaminonaphthalene) solution in 0.1 M HCl were added to the filtrate and heated at 60°C for 30 min. The piaselenol formed was extracted with 4 mL cyclohexane, vortexed for 10 s, and measured spectrophotometrically in triplicate at 378 nm using a microplate reader (Synergy H1 Hybrid Multi-Mode Reader, BioTek Instruments Inc., Winooski, VT, USA). The entrapment efficiency (EE%) was determined according to Eq 1 [28]. The selenite amount was calculated by appropriate calibration curve of free selenite (R^2^ = 0.9901).

$$EE\;(\%)=\frac{amount\;of\;active\;compound\;entrapped}{\;initial\;active\;compound\;amount}\;X\;100$$ (1)

#### Nanoparticle size, size distribution, zeta potential, and morphology

Particle size, zeta potential, and polydispersity index (PDI) were obtained using a Malvern Zetasizer NanoZS (Malvern Instruments Inc., Worcestershire, UK) at 25°C with a 633-nm laser and measurement angle of 173° for 120 continuous accumulation times. Chitosan nanoparticles (200 μg) were suspended in 2 mL of 0.2 μm-filtered deionized water and sonicated (Branson 2800 ultrasonic cleaner, 60 W, Danbury, CT, USA) for 15 min before analysis.

The morphology of the nanoparticles was analyzed using a Scanning Electron Microscopy (SEM, JEOL JCM-5700, Peabody, MA, USA) at 5 kV and magnifications ranging from 30 to 10,000x. Dried nanoparticle samples were adhered to a conductive carbon tape with a thin aluminum foil core (Nisshin EM Co., Ltd, Japan). Subsequently, samples were mounted on specimen stubs and coated with a thin film (< 20 mm) of gold and platinum layer using a sputter coater (Denton Desk V, Denton Vacuum, Moorestown, NJ, USA).

#### *In vitro* Selenium release profile

Selenium release from chitosan nanoparticles was evaluated by suspending them in phosphate buffer saline (PBS, pH 7.4) at 1 mg mL^-1^ of Se based on entrapment efficiency results. Samples were divided in 5-mL aliquots and transferred to a shaker under constant agitation at 100 rpm and 28°C in the dark. The temperature was selected to simulate the conditions observed during Nile tilapia cultivation. At incremental time intervals up to 48 h the Se released was measured. At predetermined time intervals, a sample was filtered using 0.2-μm cellulose membrane syringe filter (MilliporeSigma) and then Se content was measured using the method described in the *Entrapment efficiency* section.

#### Antioxidant activity

Radical scavenging activity was measured using the DPPH (2,2-diphenyl-1-picryllhydrazyl) radical scavenging method described by Brand-Williams et al. [29] with modifications. Briefly, Se-loaded nanoparticles (5 mg Se based on entrapment efficiency results) and equivalent mass of unloaded nanoparticles were suspended in 5 mL of deionized (DI) water and stirred at 100 rpm in the dark for 48 h at 25°C. Next, samples were filtered using 0.2-μm cellulose membrane syringe filter (MilliporeSigma), and 0.1 mL-aliquot of each sample was vigorously mixed with 3.9 mL of 0.01 mmol DPPH radical in methanol. Then, each solution was incubated for 30 min in the dark at 25°C and their absorbances were measured at 517 nm (Synergy H1 Hybrid Multi-Mode Reader). The radical scavenging activity, expressed in % scavenging ratio (*I*) was calculated using Eq 2:

$$I=\left[\frac{\left(A_{o}-A_{i}\right)}{A_{o}}\right]\;x\;100$$ (2)

Where *A*_*o*_ and *A*_*i*_ are the absorbances of the blank (0.1 mL DI water + 3.9 mL 0.01 mmol DPPH) and nanoparticle samples, respectively.

### Feeding trial experimental design

The use and care of Nile tilapia (*Oreochromis niloticus*) fingerlings and the experimental protocols were approved by the Institutional Animal Care and Use Committee at Texas A&M University (College Station, TX, USA).

#### Animal housing

The experiment was carried out at the Texas A&M Aquacultural Research and Teaching Facility, College Station, USA. Nile tilapia fingerlings (*Oreochromis niloticus*) from local commercial hatchery were used. Fish were fed a commercial diet (35% crude protein) for 2 weeks (5% of body weight daily) before the trial started. The animals were maintained in the Fish Nutrition Laboratory and stocked in a recirculation system equipped with physical and biological filters and air diffusers. The photoperiod of 12 h light:12 h dark was used and water temperature was maintained within narrow limits by conditioning the ambient air (~27°C).

Water quality was monitored three times a week throughout the experiment and maintained within suitable ranges for tilapia culture. The water quality variables analyzed are presented as follows (average ± standard deviation): temperature (27 ± 1.0°C), dissolved oxygen (6.0 ± 0.4 mg L^-1^), pH (6.9 ± 0.1), and ammonia (0.1 ± 0.02 mg L^-1^). Water temperature and dissolved oxygen were measured using a YSI ProODO meter (YSI Inc., Yellow Springs, OH, USA), and total ammonia nitrogen (TAN) was measured spectrophotometrically (Hach Inc, Loveland, CO, USA).

#### Experimental diet preparation

Five experimental diets were formulated to contain approximately ⁓36% of crude protein and ⁓14.8 MJ kg^−1^ of gross energy (S1 Table, see S1 File), which were based on a previous formulation for Nile tilapia [30]. The basal purified diets were composed of casein and gelatin as protein sources, soybean oil as the lipid source, and dextrinized starch as carbohydrate source. Except for the control diet and Se-Nano x1.5, each treatment was formulated to contain 0.5 mg of selenium per kg of diet on a d.w. basis according to the findings of previous studies on optimum dietary Se requirement of freshwater fish juveniles [31, 32]. Thus, sodium selenite (Se-Na), selenomethionine (Se-Met) as well as chitosan-Se nanoparticles (Se-Nano and Se-Nano x1.5) were used, with Se-Nano x1.5 having one and half times the amount of Se-Nano added to the diet which provided the analyzed Se levels listed on Table 3. Se-loaded nanoparticles were prepared as described in section 2.2. Selenium supplements were added to the diet at the expense of cellulose, and diets were manufactured as sinking pellets using a 3-mm die plate, stored in opaque bags and stored frozen (20°C) until used. Aliquots of each diet were ground for analysis of proximate composition.

#### Feeding trial conditions

Two hundred Nile tilapia fingerlings weighing 5.8 ± 0.8 g were stocked in 20 experimental units (tanks with 110 L of useful volume), with 10 juveniles per tank. The experiment was a completely randomized design with five treatments based on selenium sources (T1 = control, T2 = Na-Selenite, T3 = Se-Met, T4 = Se-Nano and T5 = Se-Nano x1.5) and four replicates.

Throughout the feeding trial, the experimental diets were provided twice a day (at 8:00 and 16:00 h). Animals were weighed weekly, and rations were provided according to a fixed percentage of body weight to maintain a level close to satiation without overfeeding. Uneaten feed and feces were removed (approximately 30 minutes twice a day after each meal). The feeding trial lasted 60 days.

#### Sampling procedures and performance indices

At the end of the feeding trial, fish (3–5 fingerlings) from each tank were randomly sampled and immersed in tricaine methane-sulfonate (MS-222, Western Chemical Inc, Ferndale, WA, USA) mixed with culture water to anesthetize the animals. Each fish had blood drawn from the caudal vein using heparinized syringes, and immediately euthanized with an overdose of MS-222 (~250 mg L^-1^). Fish were individually weighed and dissected for the collection of liver, kidney and muscle samples. Liver and intraperitoneal fat were excised and individually weighed to compute the hematosomatic index (HSI) and intraperitoneal fat (IPF) ratio. Liver, kidney and muscle samples were pooled separately per tank and immediately stored in -20°C to further analyze the glutathione peroxidase activity and radical scavenging activity (DPPH). Fillets were removed together with the skin and scales, which were separated afterwards to calculate the fillet yield. Three fish per tank also were euthanized to analyze the proximate composition of the whole-body.

Production performance parameters, HSI, IPF ratio, and survival rate were calculated as follows:

$$Weight\;gain\;(g,\%)=\left(\frac{final\;weight-initial\;weight}{initial\;weight}\right)\;x\;100$$ (3)

$$Hepatosomatic\;index\;(HSI,\%)=\left(\frac{liver\;weight}{wet\;body\;weight}\right)\;x\;100$$ (4)

$$Intraperitoneal\;fat\;(IPF,\%)\;ratio=\left(\frac{intraperitoneal\;fat\;weight}{body\;weight}\right)\;x\;100$$ (5)

$$Fillet\;yield\;(\%)=\left(\frac{fillet\;weight-live\;weight}{live\;weight}\right)\;x\;100$$ (6)

$$Feed\;Efficiency\;(g/g)=\frac{weight\;gain}{dry\;feed\;intake\;weight}$$ (7)

$$Survival\;rate\;(\%)=\left(\frac{final\;number\;of\;fish}{initial\;number\;of\;fish}\right)\;x\;100$$ (8)

#### Diets and whole-body composition

The diets and whole-body composition, expressed as moisture content, crude protein, total lipids, and ash were determined as described by the Association of Official Analytical Chemists [33]. Briefly, dry matter was determined by drying samples at 105°C until constant weight; ash, by incineration in a muffle furnace at 650°C for 4 h; lipid by cold extraction using the Folch method, where the lipid was washed from the samples using chloroform:methanol (2:1) as solvents, and crude protein was estimated from total nitrogen by combustion of the samples using the Dumas method. Se content in fish carcass and diets was determined by Atomic Absorption Spectrometry as described by Moreno et al. [34] with modifications. Briefly, dried muscle tissue samples and diet samples were homogenized, frozen with liquid nitrogen, and lyophilized for 12 h. Next, 83.3 mg of dried samples were digested with 0.83 mL of nitric acid and 0.33 mL of hydrogen peroxide in a microwave oven (CEM, Mattheus, NC, USA), followed by filtration (0.45 μm-nylon filter, MilliporeSigma). Then, digested samples were analyzed for ^82^Se with a 2380 Perkin Elmer atomic absorption spectrometer (Norwalk, CT, USA) using argon as the carrier gas and setting the quartz cell at 900°C [34].

#### Antioxidant activity

Antioxidant activity in fish tissue was determined according to radical scavenging activity using the method described by Thongprajukaew et al. [35] with modifications. Briefly, the tissues (muscle, liver, kidney and plasma) were homogenized in PBS buffer (4°C and pH 7.4) at 1:1 ratio and centrifuged at 10,000 x g for 20 min at 4°C. The supernatant was collected and diluted in PBS, and a 0.1 mL-aliquot of a 15-fold diluted sample was mixed with 3.9-mL DPPH radical (24 mg of DPPH in 100 mL of methanol) solution. This solution was allowed to react in the dark for 30 min, and the absorbance was measured at 517 nm (Synergy H1 Hybrid Multi-Mode Reader). The control sample consisted of PBS buffer. The radical scavenging activity (i.e., radical scavenging ratio, *I*) was calculated using Eq 2.

#### Glutathione peroxidase activity

Glutathione peroxidase activity was measured using the HT Glutathione Peroxidase Assay Kit from Trevigen (Gaithersburg, MD, USA) according to manufacturer’s protocol and described by Selvaraj et al. [36]. Briefly, tissue sample (liver, plasma, kidney and muscle) were prepared following the same procedure described above for the radical scavenging activity. The selenium-dependent glutathione peroxidase activity was tested using the assay buffer, supplemented with glutathione reductase and NADPH, and hydrogen peroxide as substrate for the oxidation of glutathione. The test was performed in a 96-well plate with each well containing a total of 350 μL mL of sample and reagents. The NADPH absorbance was measured at 340 nm (Synergy H1 Hybrid Multi-Mode Reader). The rate of reaction was estimated from the absorbance readings at the first 10 min, after the addition of hydrogen peroxide. The rate of decrease in the absorbance at 340 nm was directly proportional to the glutathione peroxidase activity in the sample. The selenium‐dependent glutathione peroxidase activity measurements were made in triplicate and expressed in nM min^-1^.

### Statistical analysis

All measurements were made at least in triplicate and results were expressed as mean ± standard deviation. Differences between variables were tested for significance using one-way analysis of variance (ANOVA) and validated for homogeneity of variances by the Brown-Forsythe test [37]. Significantly different means (p < 0.05) were evaluated using Tukey’s Honest Significant Differences (HSD) test using JMP v.13 software (SAS Institute, Cary, NC, USA).

## Results and discussion

### Nanoparticles characterization

Entrapment efficiency, size, polydispersity index, zeta potential, and antioxidant activity of the chitosan-nanoparticles results are shown in Table 1. Chitosan nanoparticles showed high encapsulation efficiency of sodium selenite (EE = 87%). It is believed that the interaction between chitosan and sodium selenite was favored by the electrostatic attraction given their opposite charges at pH ~5.0 in aqueous suspension. The effects of chitosan and sodium selenite concentrations on EE% were demonstrated by Luo et al. [23]. EE% values increased with chitosan and decreased with sodium selenite concentrations ranging from 42% to 89% [23]. In particular, Luo et al. [23] observed that the EE% values increased linearly with the increase of chitosan concentration and that the highest EE% for 0.6 mg mL^-1^ of sodium selenite was with 2.5 mg mL^-1^ chitosan, which corroborates with the high EE% result observed herein. Similar to previous studies, the presence of sodium selenite increased (p < 0.05) chitosan nanoparticle size compared to unloaded nanoparticles [23, 38], which could be directly related to the high specific volume of sodium selenite (*v* = 0.3226 cm^3^ g^-1^). Additionally, chitosan particle sizes were similar to previously reported studies that used TPP as the crosslinker, which ranged from 270 nm to 300 nm [23]. Based on PDI results, both Se-loaded and unloaded particles presented high variability of particle size. The PDI results showed significant difference among unloaded and loaded Se-chitosan nanoparticles, with a slight increase from 0.24 to 0.28. The PDI values observed in this study are in accordance with previously reported values by Luo et al. [23]. Both unloaded and Se-loaded nanoparticles presented PDI values higher than 0.1, which indicates a polydisperse system with multiple particle size population [36, 39].

**Table 1:** Particle size, polydispersity index (PDI), zeta potential, entrapment efficiency (*EE*), and radical scavenging activity ratio *(I)* of chitosan nanoparticles loaded with selenium (Se-Nano) and unloaded (control without selenium).

Treatment	Particle size (nm)	PDI	Zeta Potential (mV)	EE (%)^*^	I (%)^*^
**Se-Nano**	300.94 ± 4.19 ^a^	0.28 ± 0.02 ^a^	47.51 ± 2.94 ^a^	86.97 ± 0.02	75.36 ± 0.56 ^a^
**Unloaded**	270.86 ± 1.82 ^b^	0.24 ± 0.01 ^b^	26.56 ± 3.72 ^b^	-	-
**p-value**	0.001	0.003	0.000	-	-

^a-b^ Mean value ± standard deviation followed by different lowercase letters within the same column for each response variable are significantly different (p < 0.05) according to ANOVA and Tukey-HSD test (n = 3).

*Unloaded particles showed negligible levels of selenium and *I*.

The chitosan matrix resulted in a positive zeta potential, which is related to protonated amino groups of chitosan being exposed on the surface of nanoparticles [40]. Similar results were observed by Luo et al. [23] when using chitosan/TPP to encapsulate selenite with values ranging from 37 to 50 mV. These authors reported that the increase of chitosan concentration (0.5 to 2.5 mg mL^-1^) promoted an increase in zeta potential results, due to an increased availability of protonated NH_3_^+^ on the particle surface prepared with increased chitosan concentration; however, no results were reported for unloaded chitosan nanoparticles [23]. This superficial positive charge gives mucoadhesive properties to chitosan-based nanoparticles, making them an interesting option for drug delivery purposes, because they can interact electrostatically with the negatively charge mucosal surfaces [17, 40, 41].

The encapsulation with chitosan significantly increased the antioxidant activity of selenite. The radical scavenging activity ratio (*I*) increased from 12.00 ± 2.21% for free selenite to 75.36 ± 0.56% for Se-loaded nanoparticles. Similarly, Chen et al. [42] reported an increase of more than 80% in DPPH radical scavenging ability for Se-loaded chitosan nanoparticles compared to free sodium selenite. According to these authors, the interaction between the nanoparticles and the free radicals can be enhanced by the hydroxyl-rich chitosan surface. Basically, the DPPH scavenging method is based on the reduction of this free radical in the presence of an antioxidant compound through the donation of a hydrogen atom [43, 44]. Dumore et al. [45] suggests that Se-loaded nanoparticles are capable of promoting the reduction of the radical leading to the DPPH-H non-radical form. These authors also demonstrated the superior antioxidant activity of encapsulated selenite, using gallic acid as the encapsulant, compared to free sodium selenite [45]. The efficiency of chitosan encapsulation to increase the antioxidant activity of selenite also was demonstrated by Luo et al. [23] using hydroxyl radical scavenging method. Se-loaded nanoparticles presented 2 to 13 times higher antioxidant activity compared to equivalent concentrations of free selenite [23].

**Fig 1:**
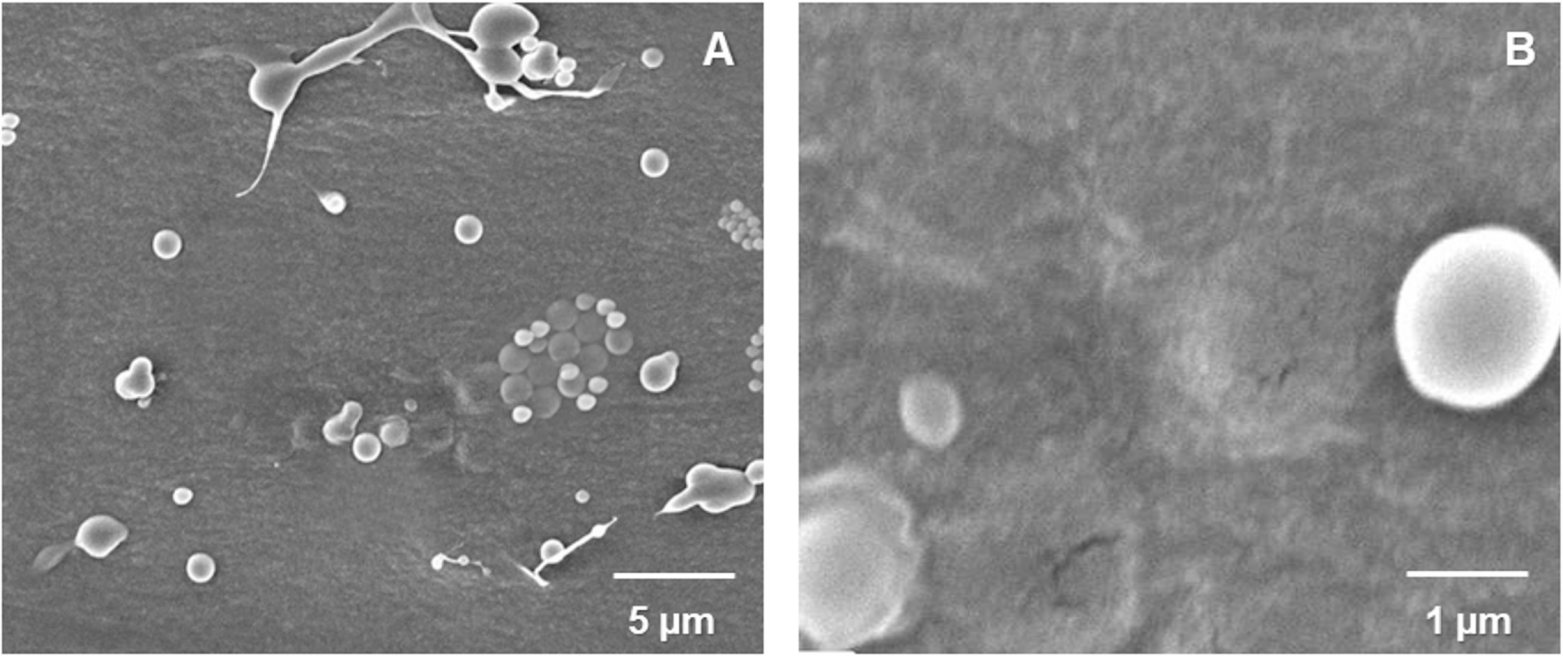
Micrographs of chitosan nanoparticles. Scanning electron microscopy (SEM) images at 5 kV of selenium-loaded chitosan nanoparticles at A) 3,000x and B) 10,000x magnification.

SEM images were obtained to characterize the surface morphology of the chitosan-based nanoparticles (Fig 1). Fig 1A and 1B show the size and surface morphology of Se-loaded nanoparticles. The Se-loaded nanoparticles presented high variability in size, as previously indicated by the PDI. All chitosan nanoparticles showed a sphere-shaped structure with smooth surface, similar to previous studies [23, 25, 46, 47].

**Fig 2:**
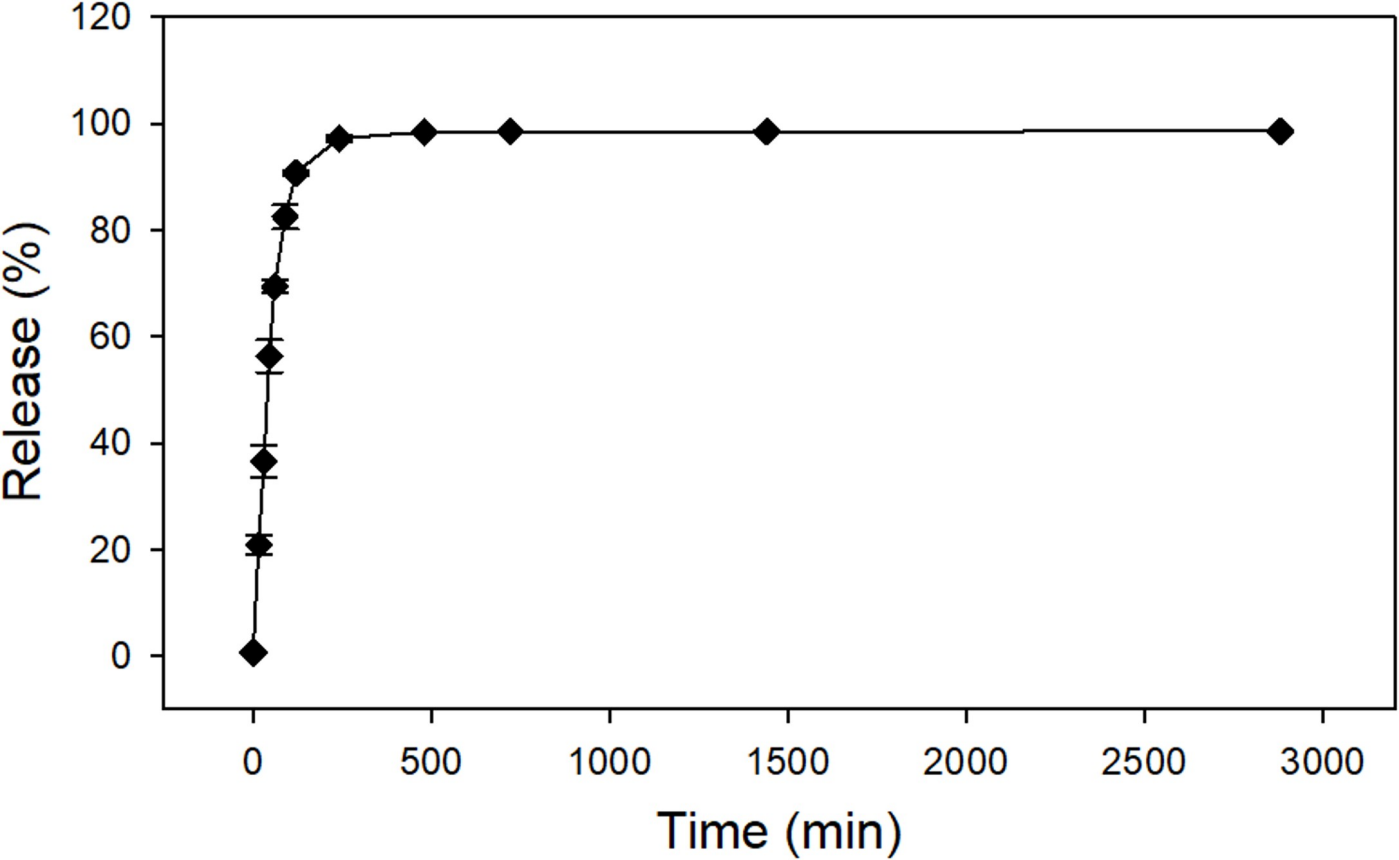
Selenium controlled release. Se released from chitosan nanoparticles over 48 h (2880 min) in phosphate buffer saline at pH 7.4 and temperature of 28°C. Data shown as mean ± standard deviation, n = 3.

Fig 2 shows the *in vitro* Se release profile from chitosan nanoparticles over 48 h in PBS at 28°C to resemble conditions of Nile tilapia cultivation. The chitosan nanoparticles presented an initial burst effect release of Se during the first 120 min (2 h), when almost 91% of the initial concentration present in the sample was released. Then, the release became gradual over the remaining test time (48 h). Subsequent to the initial burst release, the cumulative amount of selenium released increased incrementally at a reduced rate to approximately 98.6% of the selenium originally present in the nanoparticles. Similar results have been observed by other studies [23, 25, 47]. The release of a compound from polymeric matrix is a function of many variables, including diffusion through the polymer matrix, polymer erosion, compound affinity to the polymer, and swelling degree of the polymer [15, 46, 48]. As observed by the release profile, a large amount of selenium was released from the particle in a very short period of time due to its small molecular size and solubility in water. Subsequent to the initial burst effect, the release rate decreased, due to the longer distance the selenium needed to move across the polymer matrix, resulting in a steady-state release profile being observed. Consequently, the cumulative amount of selenium was kept constant for the remaining time.

### Feeding trial results

#### Fish growth parameters

Weight gain, final weight, HSI, IPF ratio, fillet yield, feed efficiency, and survival of Nile tilapia given the different Se sources are shown in Table 2. Nile tilapia weight gain was significantly improved by the presence of Se-Na and Se-Nano in diets. While supplemental Se in the diets increased the average final weight compared to control, no significant difference in final weight was observed among the different dietary treatments. Feed efficiency was significantly higher in fish fed with all Se treatments compared to the control. The survival was not significantly affected by the dietary treatments during the 60-day period with all dietary treatments showing above 97% survival.

**Table 2:** Comparison of growth performance indices of different experimental diets fed to Nile tilapia (*Oreochromis niloticus*) fingerlings for 60 days.

Treatment	Weight gain (%)	Final weight (g)	HSI^1^ (%)	IPF ratio^2^ (%)	Fillet yield (%)	Feed efficiency (g/g)	Survival (%)
**Control**	242 ± 30 ^b^	20.4 ± 2.1	2.11 ± 0.28	1.67 ± 0.81	25.72 ± 2.88	0.65 ± 0.01 ^b^	100.0 ± 0.0
**Se-Na**	346 ± 40 ^a^	26.4 ± 2.5	2.19 ± 0.39	1.85 ± 0.27	26.15 ± 5.61	0.74 ± 0.03 ^a^	97.5 ± 5.0
**Se-Met**	285 ± 70 ^b^	22.9 ± 3.8	2.43 ± 0.29	2.26 ± 0.65	26.21 ± 3.22	0.69 ± 0.06 ^a^^b^	100.0 ± 0.0
**Se-Nano**	319 ± 43 ^a^	24.9 ± 2.7	2.68 ± 0.15	1.73 ± 0.20	25.97 ± 5.72	0.71 ± 0.03 ^a^^b^	100.0 ± 0.0
**Se-Nano x1.5**	265 ± 43 ^b^	22.0 ± 2.6	2.20 ± 0.31	1.95 ± 0.33	26.73 ± 5.54	0.68 ± 0.03 ^a^^b^	100.0 ± 0.0
**p-value**	0.04	0.06	0.08	0.52	0.99	0.03	0.44

^1^ HSI: hepatosomatic index

^2^ IPF: intraperitoneal fat.

^a-b^ Mean value ± standard deviation followed by different lowercase letters within the same column for each response variable are significantly different (p < 0.05) according to ANOVA and Tukey-HSD test. n = 40 per treatment.

Se-Na: sodium selenite; Se-Met: selenomethionine; Se-Nano: chitosan selenite nanoparticles; Se-Nano x1.5: one and half time the amount of Se-Nano added. Control diets consisted of formulation listed in S1 Table of S1 File without selenium added.

The results of the present study indicate that dietary supplementation of Se in the form of Se-Na and Se-Nano significantly enhanced feed efficiency and consequently, final weight gain compared to fish fed the control diet (Table 2). Similarly, Se in an amino acid chelate organic form promoted maximum larval growth of gilthead seabream (*Sparus aurata*) [3]. Another study reported significantly higher weight gain, specific growth rate, and feed efficiency for Nile tilapia fed with Se-Met (2.06 mg kg^-1^ d.w. diet) compared to those fed with other diets (ranging from 0.0 to 14.7 mg Se kg^-1^ d.w. diet) [32]. Recently, a study by Neamat-Allah et al. [49] reported that Nile tilapia fed with nano-Se (0.7 mg kg^-1^ d.w. diet, 30–45 nm particle size) had significantly improved growth performance results compared to free Se (sodium selenite, 0.7 mg kg^-1^ d.w. diet) and control diet groups. Moreover, organic Se supplementation (up to 0.3 mg kg^-1^ d.w. of diet) showed significant improvement in feed utilization and growth performance of African catfish (*Clarias gariepinus*) [50]. Another recent study by Baldissera et al. [51] demonstrated that silver catfish (*Schilbe mystus*) fed with nanoencapsulated selenium (diphenyl diselenide-loaded nanocapsules) had significant improvement in growth performance compared to the control group in addition to improvement in antioxidant/oxidant status. Furthermore, a previous study by Zhou et al. [31] demonstrated that dietary Se nanoparticles significantly improved final weight and Se concentration in muscle of crucian carp (*Carassius auratus gibelio*) compared to Se-Met. Meanwhile, no significant difference was observed on growth performance and survival of hybrid striped bass (*Morone chrysops* x *M*. *saxatilis*) [5] fed with Se-Na or Se-Met and the control, indicating that growth response to dietary Se supplementation is dependent on fish species. HSI and IPF ratio of tilapia in the present study showed no differences among the treatments, which were similar to previous study with common carp (*Cyprinus carpio*) fed diets supplemented with organic, inorganic, or encapsulated Se [52]. Furthermore, these results corroborate those reported by Saffari et al. [52], when testing free and encapsulated Se on survival of crucian carp (*Carassius auratus gibelio)*. Similarly, no differences in survival were observed in common carp (*Cyprinus carpio*) fed with different concentrations of selenium nanoparticles (0.98–2.51 mg kg^-1^ diet d.w.) compared to those fed the control diet containing 0.43 mg Se kg^-1^ for 8 weeks [47]. Overall, the observed results demonstrate that free (Se-Na and Se-Met) or encapsulated (Se-Nano and Se-Nan x1.5) forms of selenium significantly enhanced Nile tilapia growth performance with no detrimental effects.

#### Whole-body composition

Whole-body proximate composition of Nile tilapia fed different diets for 60 days are shown in Table 3. Fish fed with Se-Nano x1.5 diet showed a lower (p < 0.05) body moisture content compared to fish fed with Se-Na but was not significantly different from the other treatments. Additionally, there was no significant difference among treatments for protein and lipid content in Nile tilapia. Fish fed with Se-Met diet presented a lower (p < 0.05) ash content compared to control, while fish fed with nanoparticle diets (Se-Nano and Se-Nano x1.5) had no significant difference in ash content when compared to free Se sources (Se-Na and Se-Met).

**Table 3:** Carcass composition (muscle) of Nile tilapia (*Oreochromis niloticus*) fed with different dietary selenium sources over a 60-day period and analyzed selenium content of experimental diets.

Treatment	Moisture (%)	Protein (%)	Lipid (%)	Ash (%)	Se content in muscle (mg kg^-1^ d.w.)	Se content in diet (mg kg^-1^ d.w.)
**Control**	70.4 ± 1.35 ^a^^b^	16.7 ± 0.62	8.47 ± 1.09	4.23 ± 0.08 ^a^	0.73 ± 0.10 ^b^	0.58 ± 0.04 ^c^
**Se-Na**	70.8 ± 0.71 ^a^	16.9 ± 0.27	7.96 ± 1.49	3.94 ± 0.20 ^a^^b^	1.08 ± 0.81 ^a^	1.00 ± 0.07 ^b^
**Se-Met**	71.6 ± 0.78 ^a^^b^	16.8 ± 0.70	7.88 ± 0.76	3.81 ± 0.14 ^a^	1.10 ± 0.76 ^a^	0.99 ± 0.02 ^b^
**Se-Nano**	70.1 ± 0.61 ^a^^b^	17.0 ± 0.18	8.58 ± 0.91	3.85 ± 0.31 ^a^	1.02 ± 0.14 ^a^	1.02 ± 0.08 ^b^
**Se-Nano x1.5**	69.4 ± 0.33 ^b^	17.0 ± 0.16	8.96 ± 1.16	4.10 ± 0.14^a^	1.05 ± 0.93 ^a^	1.50 ± 0.10 ^a^
**p-value**	0.02	0.77	0.63	0.03	<0.001	0.0071

^a-b^ Mean value ± standard deviation followed by different lowercase letters within the same column for each response variable are significantly different (p < 0.05) according to ANOVA and Tukey-HSD test, n = 40 per treatment for carcass composition and n = 3 for Se content in diet.

Se-Na: sodium selenite; Se-Met: selenomethionine; Se-Nano: chitosan selenite nanoparticles; Se-Nano x1.5: one and half times the amount of Se-Nano added. Control diets consisted of formulation listed in S1 Table of S1 File without selenium added.

Similarly, Zhou et al. [31] reported that Se nanoparticles and Se-Na diets did not affect protein, lipid, moisture, and ash content in crucian carp (*Carassius auratus gibelio)* muscle. Meanwhile, fish fed with the Se-supplemented diets showed the highest selenium content in muscle (p < 0.05) compared to fish fed the control (Table 3). There was no significant difference of Se concentration in fish muscle of those fed different Se-containing diets, which was directly related to the actual Se concentration present in the diets. The content of Se in fish muscle was similar to results reported by Lee et al. [32] for the same dietary Se concentration supplemented to Nile Tilapia (i.e., 0.55 and 1.04 mg kg^-1^ diet d.w.). According to Ashouri et al. [47], the use of dietary Se treatments did not affect the proximate muscle composition of common carp (*Cyprinus carpio*). In rainbow trout (*Oncorhynchus mykiss*), the supplementation with Se-loaded nanoparticles increased the expression of liver proteins related to aerobic energy production, which may be associated with enhancement of energy-producing pathways in the liver cells. However, no direct evidence regarding the influence of selenium on the expression of enzymes involved in amino acid metabolism was observed [53]. In contrast, Se in an amino acid chelate organic form as dietary supplements increased significantly body lipid reserves in gilthead seabream (*S*. *aurata*) compared to Se in inorganic and nanoparticle forms, which suggests that different selenium sources could differentially influence metabolic pathways and deposition mechanisms [3].

#### Glutathione peroxidase and antioxidant activities

Antioxidant activity measured via radical scavenging activity was evaluated in plasma, liver, kidney and muscle of Nile tilapia fed the five different diets (Table 4). For all tissues evaluated, the radical-scavenging activity of fish fed supplemental Se sources were higher (p < 0.05) than the control. Additionally, muscle tissue for fish fed Se-Na showed lower (p < 0.05) radical scavenging activity compared to Se-Nano x1.5 diet. Moreover, fish fed with Se-Met showed lower (p < 0.05) plasma radical scavenging activity compared to encapsulated selenium diets (Se-Nano and Se-Nano x1.5). However, an increase in dosage of Se-Nano did not increase the radical scavenging activity in fish tissue.

**Table 4:** Antioxidant activity and glutathione peroxidase activity of different fish tissue from Nile tilapia (*Oreochromis niloticus*) fed with different dietary selenium sources over a 60-day period.

	**Radical Scavenging activity (%)**			
**Treatment**	**Plasma**	**Liver**	**Kidney**	**Muscle**
**Control**	6.9 ± 0.09 ^c^	11.2 ± 0.31 ^c^	10.2 ± 0.33 ^c^	2.5 ± 0.23 ^c^
**Se-Na**	14.8 ± 0.09 ^a^	24.4 ± 1.38 ^a^^b^	22.2 ± 1.27 ^a^^b^	8.2 ± 0.16 ^b^
**Se-Met**	10.6 ± 0.10 ^b^	20.3 ± 1.11 ^b^	17.5 ± 1.10 ^b^	5.8 ± 0.14 ^b^^c^
**Se-Nano**	14.3 ± 0.17 ^a^	25.5 ± 1.16 ^a^^b^	21.9 ± 1.25 ^a^^b^	9.4 ± 0.20 ^a^^b^
**Se-Nano x1.5**	16.1 ± 0.05 ^a^	28.7 ± 1.17 ^a^	26.2 ± 1.12 ^a^	12.4 ± 0.13 ^a^
**p-value**	<0.0001	<0.0001	<0.0001	<0.0001
	**Glutathione peroxidase activity (%)**			
**Treatment**	**Plasma**	**Liver**	**Kidney**	**Muscle**
**Control**	28.1 ± 1.12 ^d^	79.6 ± 3.51 ^d^	57.5 ± 2.16	115.9 ± 3.42
**Se-Na**	75.4 ±3.70 ^b^	148.2 ± 7.24 ^c^	31.5 ± 1.15	79.2 ± 3.14
**Se-Met**	55.4 ± 2.34 ^c^	137.0 ± 5.10 ^c^	49.9 ± 2.35	107.7 ± 3.28
**Se-Nano**	85.1 ± 3.08 ^b^	188.5 ± 13.12 ^b^	40.2 ± 2.17	86.1 ± 3.13
**Se-Nano x1.5**	116.4 ± 6.35 ^a^	233.0 ± 18.85 ^a^	52.9 ± 2.24	112.6 ±3.15
**p-value**	<0.0001	<0.0001	0.53	0.23

^a-b^ Mean value ± standard deviation followed by different lowercase letters within the same column for each response variable are significantly different (p < 0.05) according to ANOVA and Tukey test. n = 40 per treatment.

Se-Na: sodium selenite; Se-Met: selenomethionine; Se-Nano: chitosan selenite nanoparticles; Se-Nano x1.5: one and half times the amount of Se-Nano added. Control diets consisted of formulation listed in S1 Table of S1 File without selenium added.

A significant effect of selenium sources on antioxidant defense systems of Nile tilapia was observed in the present study (Table 4). Organs such as liver and kidney are equipped with antioxidant defense systems including glutathione peroxidase, catalase, and superoxide dismutase, which are essential for radical scavenging activity, regulating the presence of free radicals (i.e., reactive oxygen species) [54, 55]. It is commonly known that organic selenium such as Se-Met and selenocysteine has higher bioavailability than inorganic sources such as Se-Na to both terrestrial animals as well as aquatic species, even though fish can uptake inorganic selenium from water via their gills [56]. Se-Met bioavailability occurs through Na^+^-dependent neutral amino acid transport and seems more effective than Se-Na absorption by passive diffusion [3, 57, 58]. The results observed here indirectly indicate that nanoencapsulation of Se-Na significantly increased its bioavailability as observed by the responses of radical scavenging and glutathione peroxidase activities in different fish tissues. Our results corroborate previous studies which have demonstrated higher bioavailability of Se nanoparticles, its high effectiveness in preventing oxidative stress, and relatively low toxicity compared to other Se forms in several fish species [59–62]. Recently, Saffari et al. [63] showed that dietary Se nanoparticles acted more efficiently in growth performance and antioxidant defense system of common carp (*Cyprinus carpio*) than organic and inorganic sources of Se. Our results indicate that the use of chitosan with TPP to encapsulate Se-Na enhanced bioavailability of Se-Na, which might be due to particles being positively charged and chitosan’s muco-adhesive properties [23, 40, 41], consequently assisting in Se delivery.

Hepatic and plasmatic glutathione peroxidase activity were the highest (p < 0.05) in fish fed with diets containing selenium sources and lowest (p < 0.05) in fish fed the control diet (Table 4). These results were expected as Se plays an important role in activating antioxidant defense systems as it forms selenocysteine, which is a component of the enzyme glutathione peroxidase’s active center [64]. Glutathione peroxidase catalyzes the reduction reactions of hydrogen peroxide to water and oxygen as well as fatty acid hydroperoxides to fatty acid alcohols and oxygen at the cytosol and mitochondrial matrix [65]. Furthermore, this element is part of other active selenoproteins such as the enzyme type 1 iodothyronine 5’-deiodinases that scavenge iodine residue to regulate hormone metabolism [31, 66]. These Se functions might be directly correlated with the Se response on growth performance of Nile tilapia, as reported previously for common carp (*Cyprinus carpio*) by Ashouri et al. [47]. Additionally, fish fed diets containing encapsulated selenium (Se-Nano and Se-Nano x1.5) showed higher (p < 0.05) glutathione peroxidase activity than free selenium sources (Se-Na and Se-Met) for plasma and liver tissues. The highest (p < 0.05) hepatic and plasmatic glutathione peroxidase activities were observed in fish fed with Se-Nano x1.5 diet. These findings indicate the benefits of using Se-Nano supplemented diets and its effect in enhancing Se bioavailability. No differences were found among the different diets for glutathione peroxidase activity in the muscle or kidney tissues. Contrary to previous studies by Naderi et al. [53] and Saleh et al. [67] that used commercially available Se nanoparticles (Nano-Se from Iranian Nanomaterials Pioneer, and Sel-Plex from Alltech), the positive effect of Se nanoparticles on stress resistance showed to be directly proportional (p < 0.05) to the Se content (i.e., Se-Nano and Se-Nano x1.5) present in the diet, which demonstrates the importance of the encapsulant material on the Se bioavailability. Both glutathione peroxidase and radical scavenging activities indicated a protective effect of Se-Nano in fish tissues against oxidative damage.

## Conclusions

Encapsulation of sodium selenite in chitosan matrix was presented as a simple and cost-effective method to produce nanoparticles with high entrapment efficiency, small size, *in vitro* antioxidant activity, and controlled release properties. Overall, chitosan nanoparticles containing selenite showed to be a great alternative to protect and delivery selenium during fish production as components of fish diet supplements. The dietary supplementations with different Se sources (free and encapsulated selenium) lead to significant improvements in final weight and feed efficiency of Nile tilapia fingerlings. Conversely, Se sources had no significant effect on muscle protein and lipid content. In addition, selenium chitosan nanoparticles (Se-Nano and Se-Nano x1.5) were significantly more effective than free Se-Na and Se-Met to prevent oxidative stress and improved antioxidant activity in tissues of Nile tilapia fingerlings, as originally hypothesized. Results suggest that Se-Nano is an efficient source for providing dietary Se to Nile tilapia fingerlings. These chitosan selenite nanoparticles can have a wide range of applications with use in multiple commercial fish productions. Additionally, these chitosan nanoparticles have large potential to encapsulate other dietary supplements for controlled delivery and bioavailability. Overall, these chitosan selenite nanoparticles have the potential to improve fish production in commercial aquaculture.

## Supporting information

S1 File (DOCX)
