# Supplementary material for: Delivery of selenium using chitosan nanoparticles: Synthesis, characterization, and antioxidant and growth effects in Nile tilapia (Orechromis niloticus)
Source: PLoS One. 2021 May 18;16(5):e0251786. doi: 10.1371/journal.pone.0251786 (PMC8130939; doi:10.1371/journal.pone.0251786)
Supplement: S1 File — (DOCX) [file pone.0251786.s001.docx]

**S1 File**

**Delivery of selenium using chitosan nanoparticles: synthesis, characterization, and antioxidant and growth effects in Nile tilapia (Orechromis niloticus)**

Juliana M. Araujo^1^, Rodrigo Fortes-Silva^1,2,#a&*^, Cícero C. Pola^3^, Fernando Y. Yamamoto^4^, Delbert M. Gatlin III^4&*^, Carmen L. Gomes^3&*^

^1^ Department of Animal Science and Veterinary Medicine, Federal University of Bahia, Salvador, Bahia, Brazil.

^2^ Laboratory of Feeding Behavior and Fish Nutrition, Center of Agricultural, Environmental and Biological Sciences, Federal University of Bahia, Cruz das Almas, Bahia, Brazil.

^3^ Department of Mechanical Engineering, Iowa State University, Ames, Iowa, United States of America.

^4^ Department of Wildlife and Fisheries Sciences, Texas A&M University, College Station, Texas, United States of America.

^#a^ Current Address: Department of Animal Science, Federal University of Viçosa, Viçosa, Minas Gerais, Brazil.

*Corresponding author

E-mail: fortesrs@yahoo.com.br (RF-S), d-gatlin@tamu.edu (DMG), carmen@iastate.edu (CLG)

^&^RF-S, DMG, and CLG are Joint Senior Authors

**
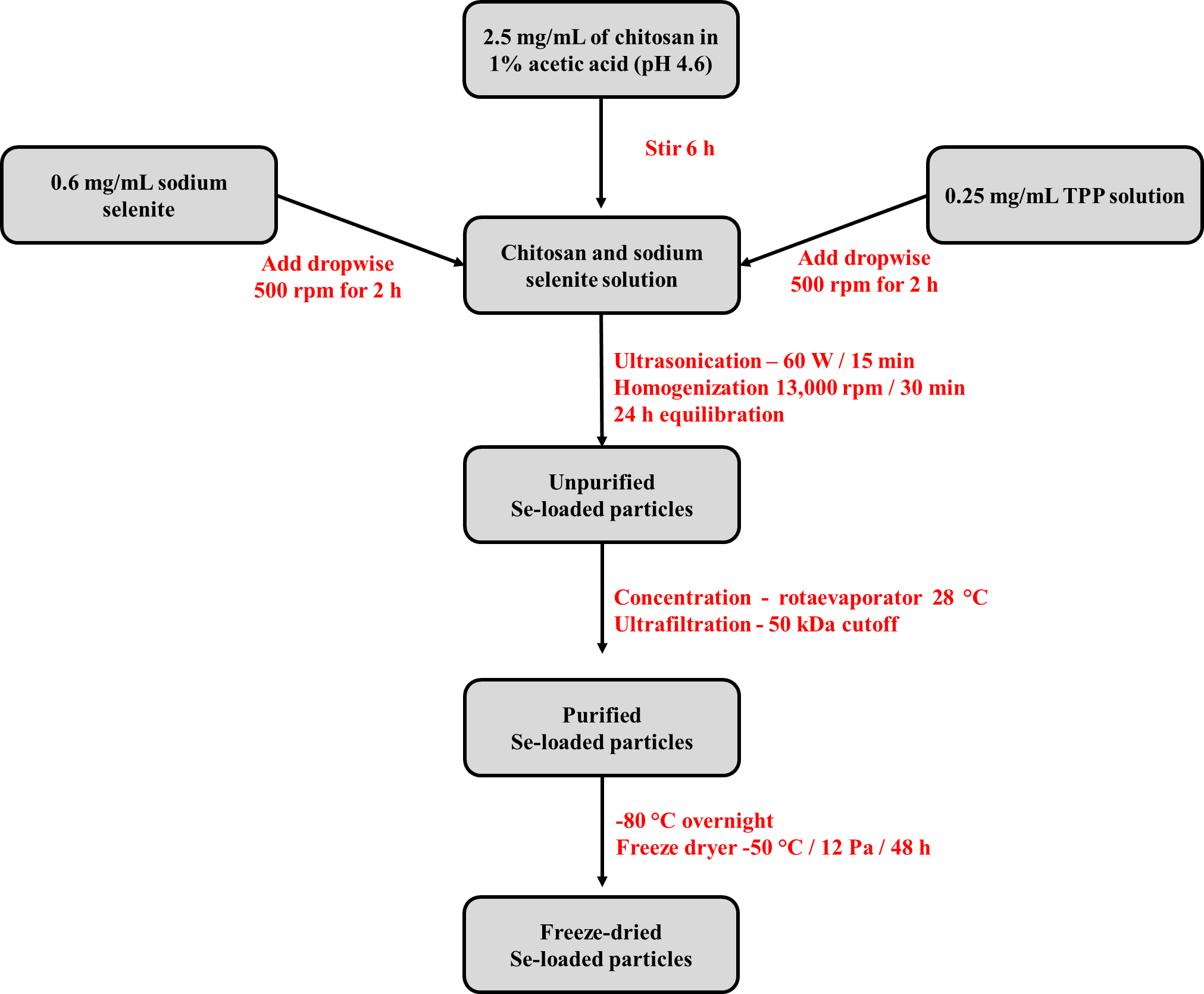
**

**S1 Fig.** **Selenium-loaded chitosan nanoparticles production.** Schematic diagram of Selenium-loaded chitosan nanoparticles synthesis.

**S1 Table. Formulation and proximate composition of the experimental diets (dry weight basis).**

|  | **Treatment** | | | | |
| --- | --- | --- | --- | --- | --- |
| **Ingredients**  **(g 1000 g^-1^ d.w.)** | **Control** | **Na-Selenite** | **Se-Met** | **Se-Nano** | **Se-Nano x1.5** |
| **Casein** | 329.44 | 329.44 | 329.44 | 329.44 | 329.44 |
| **Gelatin** | 37.98 | 37.98 | 37.98 | 37.98 | 37.98 |
| **Soybean oil** | 52.52 | 52.52 | 52.52 | 52.52 | 52.52 |
| **Dextrinized starch** | 255.95 | 255.95 | 255.95 | 255.95 | 255.95 |
| **Vitamin premix** | 29.36 | 29.36 | 29.36 | 29.36 | 29.36 |
| **Mineral premix** | 44.34 | 44.34 | 44.34 | 44.34 | 44.34 |
| **Arginine** | 4.64 | 4.64 | 4.64 | 4.64 | 4.64 |
| **Carboxymethylcellulose** | 20.29 | 20.29 | 20.29 | 20.29 | 20.29 |
| **Cellulose** | 225.48 | 215.01 | 215.01 | 215.01 | 215.01 |
| **Se-Na^*^** | - | 10.47 | - | - | - |
| **Se-Met^*^** | - | - | 10.47 | - | - |
| **Se-Nano^*^** | - | - | - | 10.47 | - |
| **Se-Nano x1.5^*^** | - | - | - | - | 21.94 |
| **Proximate composition (% d.w.)** |  |  |  |  |  |
| **Moisture** | 6.4 | 6.6 | 6.5 | 6.6 | 6.6 |
| **Protein** | 35.8 | 35.9 | 35.9 | 35.9 | 35.9 |
| **Lipid** | 9.1 | 9.9 | 9.2 | 9.3 | 9.3 |
| **Ash** | 8.1 | 8.6 | 8.7 | 8.9 | 9.0 |
| **Energy (MJ kg^-1^)** | 14.30 | 14.88 | 14.78 | 14.87 | 14.87 |

Vitamin premix (g kg^-1^): ascorbic acid, 50; DL- calcium pantothenate, 5; choline chloride, 36.2; inositol, 5; menadione sodium bisulfite, 2; niacin, 5; pyridoxine.HCl, 1; riboflavin, 3; thiamine mononitrate, 0.5; DL-alpha-tocopherol acetate (250 IU/g), 8; vitamin A palmitate (500,000 IU/g), 0.2; biotin, 0.5; folic acid, 1.8; vitamin B12, 0.02; cholecalciferol (40 IU/μg), 0.02; cellulose, 971.76.

Mineral premix (g): Ca(H_2_PO_4_) 2H_2_O, 136; Ca(C_6_H_10_O_6_) 5H_2_O, 348.5657; FeSO_4_ 7H_2_O, 5; MgSO_4_ 7H_2_O, 132; K_2_HPO_4_, 240; NaH_2_PO_4_ H_2_O, 88; NaCl, 45; AlCl_3_ 3H_2_O, 0.084; KI, 0.15; CuSO_4_ 5H_2_O, 0.5; MnSO_4_ H_2_O, 0.7; CoCl_2_ 6H_2_O, 1; ZnSO_4_ 7H_2_O, 3.

^*^Se-Na: sodium selenite; Se-Met: selenomethionine; Se-Nano: chitosan selenite nanoparticles; Se-Nano x1.5: Times one and a half the amount of Se-Nano added. A Se-premix was prepared with 100 g of celulose for all Se diets, which corresponded to 10.47 g kg^-1^ added to the diet formulation to obtain supplement 0.5 mg Se per kg of diet (the analyzed Se levels are listed on Table 3).°
